# Adaptation and validation of a Rwanda-focused version of the Alcohol Use Disorder Identification Test (AUDIT)

**DOI:** 10.1371/journal.pone.0316993

**Published:** 2025-02-25

**Authors:** Noah Rosenberg, Mia Buono, Vincent Ndebwanimana, Joseph Niyonzima, Roland C. Merchant, Aly Beeman, Catherine A. Staton, Joao Ricardo Nickenig Vissoci

**Affiliations:** 1 Department of Emergency Medicine, Duke University School of Medicine, Durham, North Carolina, United States of America; 2 Department of Emergency Medicine, University of Botswana, Gaborone, Botswana; 3 Duke Global Health Institute, Duke University, Durham, North Carolina, United States of America; 4 University of Rwanda, Kigali, Rwanda; 5 Icahn School of Medicine at Mount Sinai, New York, New York, United States of America; 6 Alpert Medical School, Brown University, Providence, Rhode Island, United States of America; University of the Witwatersrand Johannesburg Faculty of Health Sciences, SOUTH AFRICA

## Abstract

In Rwanda, alcohol use disorder (AUD) is estimated to affect 7% of the population. The Alcohol Use Disorder Identification Test (AUDIT) is an excellent screening instrument for AUD, but a Rwanda-focused version previously was unavailable. Our objective was to develop a Rwanda- focused AUDIT and evaluate its psychometric properties.

The English AUDIT was adapted to the Rwandan language through translation and back- translation by a panel of native English and Kinyarwanda speakers. Random sampling was used to recruit participants from the emergency department, outpatient clinics, and inpatient wards at a tertiary care center in Rwanda, excluding those < 18 years old, declining to participate, unable to provide consent, or when participation would interfere with care. Participants completed the Rwanda-focused AUDIT using an audio computer-assisted self-interviewing format. Internal structure was assessed using one-, two- and three-dimensional models of fit and confirmatory factor analysis (CFA), assessed by Chi-square (χ2), Root Mean Square Error of Approximation (RMSEA), Tucker-Lewis index (TLI) and comparative fit index (CFI).

Of 775 patients assessed for enrollment, 7% were unable to provide consent, 12% declined to participate, 2% could not participate because it would disrupt their medical care, and 1.3% dropped out, leaving 614 included for analysis. Of the 614, the majority were male (61%), married (53%) and had only primary education (65%). Their ages were: 33% 18-30, 43% 31-50, and 25% > 50 years-old. Factor loading for the AUDIT CFA model was between 0.62 and 0.96 for all items. Model fit indices included χ2 of < 0.001, RMSA of 0.061 (0.049 - 0.073), TLI of 0.994, and CFI of 0.995. Reliability statistics included Cronbach’s alpha at 0.91 (0.90 – 0.92), Omega 6 at 0.948 and composite reliability at 0.977.

The Rwanda-focused AUDIT showed excellent performance for measures of internal structure with high factor loading on CFA and model fit indices meeting traditional parameters of RMSEA < 0.08, TLI > 0.90, and CFI > 0.95. In this context, χ2 should ideally be > 0.05, however a relatively large sample, such as ours, tends to depress the number. All reliability statistics were above 0.90, indicating strong internal consistency. These findings support the reliability of this screening instrument. Further research should focus on the development of brief interventions for those who screen positive.

## Introduction

Harmful alcohol use results in over 3 million deaths and a loss of 132.6 million disability- adjusted life years (DALYs) annually [1]. The association between AUD and numerous adverse health consequences has been well established, including road traffic injury, HIV medication non-adherence, intimate partner violence, liver disease, hypertension, and cancer [2].

Africa is disproportionately affected by the negative health consequences of alcohol. The age-standardized alcohol-attributable death rate in Africa is the highest anywhere in the world at 70.6 deaths per 100,000 people [1]. In Rwanda, a country of over 14 million people, AUD affects approximately 7% of the population (12.2% for men), compared to 3.7% for the World Health Organization (WHO) African region [3]. Alcohol consumption in Rwanda per capita is among the highest in Africa at 9.0 liters of pure alcohol consumed per person per year, as compared to 6.3 for the African region [1]. Of the 10 top causes of death in Rwanda, three are linked to alcohol, including road traffic injury, HIV, and liver disease [4]. As a result of the growing alcohol-related harm in the region, the Rwandan Ministry of Health has identified reducing alcohol-related harm as a national priority [5].

On a global level, the majority of people who have AUD remain undiagnosed [6]. Screening tools are crucial to assess and identify problematic alcohol use, thereby helping to combat the associated morbidity and mortality. Excellent screening tools exist, including the Alcohol Use Disorders Identification Test (AUDIT) [7]. The AUDIT was developed by the WHO to assess three domains, including alcohol use, dependence, and harmful behavior [8]. It consists of 10 questions, has been translated into multiple languages, and validated in many populations, including high- and low-income settings (Sweden [9], Philippines [10], Iran [11], Japan [12]). Validation studies within sub-Saharan Africa have been conducted (Nigeria [13], (Tanzania [14], Zambia [15], and Mozambique [16]), but are more limited than other global regions. The AUDIT’s overall score, ranging from 0-40, is a summation of all questions. Each question is scored on a 5-point Likert-type scale with point values ranging from 0 to 4. Higher values in each dimension mean an increasing probability of alcohol misuse and dependency, with total scores greater than or equal to 8 suggesting harmful use [8].

Previous studies in Rwanda have used versions of the AUDIT translated into the Kinyarwanda language for screening [17–20]. However, no methodology for cultural and linguistic adaptation, validation metrics, or translated versions have been published. Ninety-nine percent (99%) of the 12 million Rwandans speak Kinyarwanda, but fewer than 10% speak any European language [21,22]. Kinyarwanda is also spoken in neighboring countries, leading to an estimated 20 million total Kinyarwanda speakers in the region [22]. No validated tool exists for AUD screening in Kinyarwanda. Thus, a validated version of the AUDIT in the local language is needed to address the growing alcohol-related harm.

Although there is a need for a validated AUD screening instrument, current screening practices present challenges in the Rwandan context. These challenges include limited literacy and a lack of private clinical spaces in healthcare settings. An estimated 32% of Rwandans have limited literacy [23], and this may be higher in subgroups at higher risk for AUD. Thus, self- administered, written versions of AUDIT are not feasible. In addition, patients often occupy crowded wards and clinics, making it difficult to maintain confidentiality when a survey is administered by face-to-face interview. To address these challenges, we proposed the use of an Audio Computer-Assisted Self-Interviewing (ACASI) technique. The ACASI involves a computer interface and audio-recorded text [24] and allows patients with limited literacy to complete the screening instruments with headphones and a tablet computer. Research has shown that participants screened using an ACASI are more likely to answer sensitive questions compared to a face-to-face interview [25]. ACASI has been used successfully in similar settings [26], suggesting it is the ideal method to address the current screening challenges in Rwanda.

In this manuscript, we describe the linguistic and cultural adaptation of a Rwanda- specific version of AUDIT, delivered by ACASI. We further report on our assessment on the tool’s validity, reliability, and internal structure in multiple healthcare settings in Rwanda, including inpatient wards, outpatient clinics, and emergency departments (EDs).

## Methods

### Translation and cultural adaptations

Research assistants (RA) were selected through a standardized application process. RA positions were advertised through the medical community. Of those who applied, credentials were reviewed and a subset were interviewed for medical background, prior research experience, and organizational and communication skills. Once selected, investigators conducted on-the-job training for study protocols and directly observed performance at intervals to ensure adherence to standards. All RAs were fluent in both English and Kinyarwanda and had at least an undergraduate degree. (one paramedic, one nurse, and one with an IT background).

All text from the English AUDIT instrument was translated by a professional Kinyarwanda interpreter. A three-judge panel, consisting of two bilingual native Kinyarwanda speakers and one native English speaker, including a doctor, a nurse, and a layperson, back- translated each item from Kinyarwanda to English. The panel then compared the back-translated version to the original English and assessed for discrepancies. Discrepancies were recorded and resolved by consensus and the Kinyarwanda version was revised accordingly.

RAs visited grocery stores, bars, and beverage distributors in the Kigali area and recorded the names and alcohol content of available alcoholic drinks. A panel, including 5 native Rwandese, generated an additional list of names used for locally available homebrewed alcoholic drinks. The resulting list of commonly available alcoholic drinks in Rwanda was organized by alcohol content, using estimates for homebrew found in previous literature [27], and divided into categories of approximately 5%, 7%, 12%, and 40%, corresponding to the alcohol content categories of the original AUDIT standard drinks reference. A representative drink and corresponding image were selected for each category and incorporated with the final Kinyarwanda text to form the Rwanda-focused standard drinks reference.

During translation and adaptation, fifteen [15] words or phrases were identified that were ambiguous or inaccurate. Thematic codes were generated including: 1) “not in colloquial usage” for words or phrases that were technically correct but poorly understood by participants, 2) “ambiguous meaning” for those open to multiple interpretations, and 3) “wrong emphasis” for those that contained an emphasis in meaning that was contrary to the intention of the original English version. Examples illustrating the original English, first Kinyarwanda translation, back translation, problem identified, and final Kinyarwanda version are provided in Table 1.

**Table 1:** AUDIT linguistic adaptation from English to Kinyarwanda.

Text from English AUDIT	First Kinyarwanda translation and English back- translation	Problem with first translation identified	Final Kinyarwanda translation
**Word or phrase not in colloquial usage**			
Alcoholic beverages	ibinyobwa bisindisha = alcoholic drink	Correct formal translation but not understood by participants.	Amacupa = Bottles/drink
You found that you	Ukisanga = to find yourself	Correct formal translation but not understood by participants.	Ukiyumva = to feel yourself
**Word or phrase ambiguous**			
Typical day	Mu minsi isanzwe = ordinary days	This phrase would only mean Monday-Friday, not including Saturday or Sunday	Ubusanzwe = usually
In the last 12 months”	Mwaka ushize = the last year	“Year” can refer to calendar year rather the prior 12 months	Mezi 12 ashize = The last 12 months
**Word or phrase contains wrong emphasis**			
What was normally expected from you	Gukora ibyo wari witezweho = did you do what you were expected to do	Phrase was changed to emphasize one’s duties and responsibilities rather than usual activities	Kuzuza inshingano zawe = to fulfill your duties or responsibilities

The final Kinyarwanda text for the Rwanda-focused AUDIT was audio-recorded by a native speaker and formatted for delivery via the ACASI format, using a software platform developed by ACASI LLC. The program was loaded on tablet computers with attached headphones to provide audio privacy.

### Instrument evaluation

Participant recruitment took place in Kigali (capital city of Rwanda, with an estimated population of 1.2 million) at the University Teaching Hospital of Kigali (UTH-K). The hospital is a tertiary care center and is the University of Rwanda’s (UR) primary teaching hospital. The hospital has 576 inpatient beds and provides care for an annual volume of 22,000 patients in the ED and 37,000 in the outpatient clinic. Potential participants were selected from the ED, inpatient wards, and outpatient clinics based on a random number generator and invited to participate. Those who were < 18 years old, unable to provide consent, declined to participate, or for whom participation would disrupt medical care were excluded from the study. Participants were offered 2000 Rwandan Francs (approximately 2 United States dollars) in compensation for their time upon completion of the survey.

Pilot testing of the instrument which aimed to assess the question quality, coherence of language, and content, was conducted with a group of 34 patients selected by convenience sampling. RAs administered the instrument to participants verbally from a paper version and interviewed them regarding their understanding of each item. Interviewers used techniques from cognitive interviewing including “think-aloud” and verbal probing, described elsewhere [28].

Potentially inaccurate or ambiguous words or phrases were noted by RAs, reviewed by the panel of judges, and modifications made where necessary. Survey responses from the pilot group were excluded from statistical analysis.

Following revisions, evaluation of the final instrument was conducted in the ACASI format. A target sample size of 600 was determined based on recommendations of at least 5 to 15 subjects per item of the instrument, allowing for subgroup analysis [29]. Participants completed the Rwanda-focused AUDIT instrument in ACASI format on tablet computers with headphones. Research assistants were nearby to assist as desired by participants, but otherwise unable to see or hear participant’s responses. Responses were saved under a unique identifier in the ACASI software memory. Data were later transferred to a secure server and uploaded to R studio for analysis.

### Data analysis

#### Construct validity.

The internal structure of the AUDIT was evaluated using confirmatory factor analysis (CFA). Although the AUDIT was originally developed to cover three dimensions (alcohol use, dependence, and harmful behavior), it has also been used as a two-dimensional model (aggregating dependence and harm subscales) [10] and, most frequently, as a one-dimensional scale [30]. We evaluated three, two, and one-dimensional models. CFA model adequacy was assessed using Chi-square (χ2) with P-value and degrees of freedom (Df), Root Mean Square Error of Approximation (RMSEA), Tucker-Lewis index (TLI), and comparative fit index (CFI). These indices address how closely the model fits the data. Acceptable fit is indicated by RMSEA <0.08, TLI,>0.9, and CFI >  0.95 [31]

#### Concurrent validity.

The Diagnostic and Statistical Manual of Mental Disorders (DSM-5) is the current gold standard for assessing harmful alcohol use. DSM-5 AUD severity scores have previously been shown to correlate with AUDIT scores strongly [32,33]. The DSM-5 provides rigorous definitions of AUD which is assessed on a continuum of symptom agreement: mild (2-3 symptoms), moderate (4-5 symptoms), and severe ( ≥ 6 symptoms) [33]. We assessed validity by correlating AUDIT and DSM-5 gold standard scales and comparing AUDIT scores among drinkers to non-drinkers, hypothesizing significantly lower AUDIT scores among non-drinkers.

#### Internal reliability.

We assessed internal reliability using Cronbach’s alpha, composite reliability (CR), and McDonald’s Omega. Values of > 0.9 indicate excellent reliability for Cronbach’s alpha and McDonald’s omega; values > 0.8 indicate excellent reliability for CR but values > 0.95 can indicate some redundancy [34].

### Ethical statement

The study was approved by the Ethics Committee of the University Teaching Hospital of Kigali (EC/CHUK/030/2020), Kigali, Rwanda, and by the Institutional Review Board of Lifespan Incorporated, Providence, Rhode Island, United States (IRB1574816-1).

## Results

### Participant recruitment and retention for the instrument evaluation

**Fig 1:**
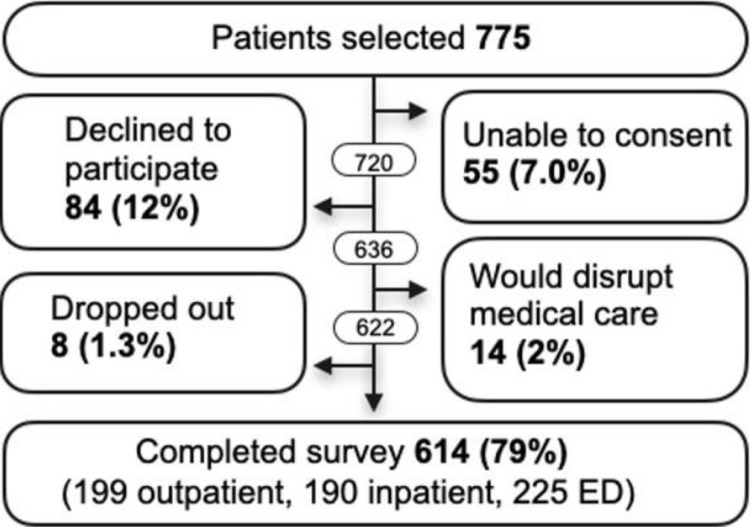
Participant recruitment and retention.

Seven hundred and seventy-five (775) patients were selected from the UTH-K ED, inpatient wards, and outpatient clinics and evaluated for participation in the instrument evaluation. Fig 1 depicts the participant recruitment and enrollment for the study. Of the 84 who declined to participate, 35 (42.7%) did so because of too much physical discomfort due to their medical condition, 22 (26.8%) did not wish to provide a reason, 16 (20%) stated that they had insufficient time to participate, 5 (6.1%) cited confidentiality concerns, and 4 (4.9%) felt uncomfortable using the touchscreen or headphones. Of the 8 participants who dropped out before completing the survey, 7 did so because they said the survey was too long and 1 did not like the headphones.

### Sample characteristics

Participants were patients in the ED (37%), outpatient clinic (32%), and inpatient ward (31%). Most of the participants were male, 31-50 years-old, classified by the Rwandan government as having middle-low socioeconomic status, married, and had limited primary education Table 2. Nearly a third of participants reported any lifetime usage of alcohol, and more than one-fifth had an AUDIT score of 8 or more points, suggesting harmful alcohol use on the AUDIT 0–40-point scoring scale.

**Table 2:** Study population demographics by clinical setting.

Demographics by Population Type	Overall, N = 614 (%)	Inpatient Ward, N = 190 (%)	Outpatient Clinic, N = 199 (%)	Emergency Department, N = 225 (%)
**Sex**				
Male	377 (61%)	133(70%)	103 (52%)	141 (63%)
Female	237 (39%)	57 (30%)	96 (48%)	84 (37%)
**Age Category (years)**				
18-30	201 (33%)	56 (29%)	61 (31%)	84 (37%)
31-50	261 (43%)	82 (43%)	86 (43%)	93 (41%)
>50	152 (25%)	52 (27%)	52 (26%)	48 (21%)
**Socioeconomic Status**				
High	84 (14%)	26 (14%)	26 (13%)	32 (15%)
Mid-High	222 (37%)	73 (39%)	75 (38%)	74 (34%)
Mid-Low	286 (48%)	82 (44%)	94 (48%)	110 (50%)
Low	7 (1%)	4 (2%)	0 (0%)	3 (1%)
No Response	15 (2%)	5 (2%)	4 (2%)	6 (2%)
**Highest Educational Attainment**				
No Formal Education	71 (12%)	23 (12%)	26 (13%)	22 (9.8%)
Some Grade School	387 (63%)	135 (71%)	114 (57%)	138 (61%)
Completed High School	156 (25%)	32 (17%)	59 (30%)	65 (29%)
**Marital Status**				
Not Married	288 (47%)	82 (43%)	81 (41%)	125 (56%)
Married	326 (53%)	108 (57%)	118 (59%)	100 (44%)
**Alcohol use**				
AUDIT ≥ 8	129 (21%)	37 (19%)	33 (17%)	59 (26%)
Lifetime Alcohol Use	194 (32%)	63 (33%)	64 (32%)	67 (30%)
Last 3 Month Alcohol Use	77 (13%)	20 (11%)	23 (12%)	34 (15%)

#### Construct validity.

**Fig 2:**
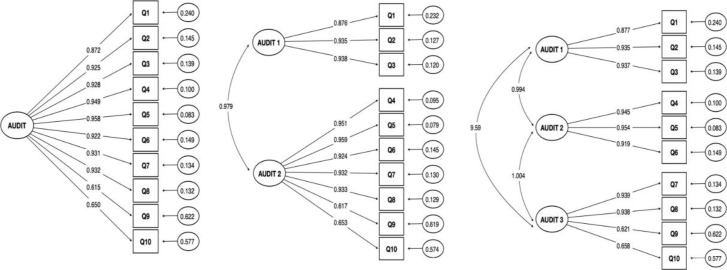
Factor loading diagram for one, two, and three-dimensional AUDIT model.

CFA of the one-dimensional AUDIT model showed items with factor loadings ranging from 0.615 to 0.959, for the two-dimensional model factor loading ranged from 0.617 to 0.959, and for the three-dimensional model from 0.621 to 0.954 (Fig 2). Model fit for our one- dimensional AUDIT model indices included χ2 of < 0.001, RMSA of 0.061 (0.049 - 0.073), TLI of 0.994, and CFI of 0.995. Model fit for our two-dimensional AUDIT model indices included χ2 of < 0.001, RMSA of 0.061 (0.048 - 0.073), TLI of 0.994, and CFI of 0.995. Model fit for our three-dimensional AUDIT model indices included χ2 of < 0.001, RMSA of 0.060 (0.047 - 0.073), TLI of 0.994, and CFI of 0.996. For the one-dimensional model, reliability statistics included Cronbach’s alpha at 0.91 (0.90 – 0.92), Omega at 0.948, composite reliability at 0.977, and average extracted variance of 0.868 for the one-dimensional model and similar for two- and three-dimensional models Table 3.

**Table 3:** Confirmatory factor analysis model fit indicators.

Model	χ2	df	RMSEA	CFI	TLI	Average Extracted Variance
AUDIT1-Factor	0.0	35	0.061	0.995	0.994	0.868
AUDIT 2- Factor	0.0	34	0.061	0.995	0.994	0.872
AUDIT 3- Factor	0.0	32	0.060	0.996	0.996	0.872

#### Concurrent validity.

**Fig 3:**
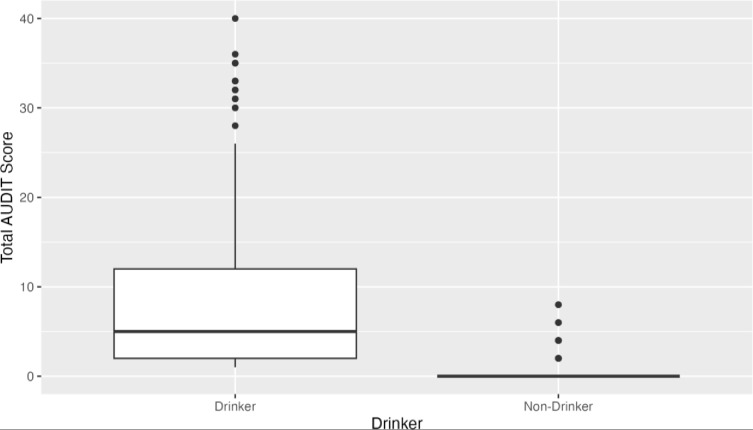
AUDIT score by drinker vs. non-drinker status.

**Fig 4:**
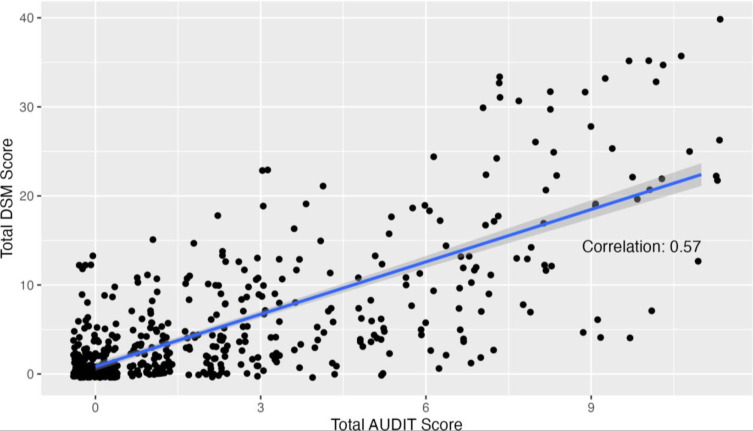
Correlation between AUDIT score and DSMALC score and known group comparison.

Group comparisons showed lower AUDIT scores for non-drinking participants versus those who have consumed alcohol in the past year, as anticipated (Fig 3). Further specificity and sensitivity analysis comparing AUDIT to the gold standard DSM Mild scores showed adequate specificity and sensitivity of the AUDIT tool (Fig 4).

#### Internal reliability.

For the one-dimensional and two-dimensional AUDIT models, all reliability values were above 0.80, and reliability values were considered adequate. For the three-dimensional model, reliability values were above 0.70 Table 4.

**Table 4:** Model reliability.

Model	Cronbach’s Alpha	Omega	Composite Reliability
AUDIT 1-Factor	0.918	0.948	0.977
AUDIT 2-Factor	0.891	0.924	0.957
AUDIT 3-Factor	0.733	0.957	0.888

## Discussion

To our knowledge, this study is the first to perform a systematic cultural and linguistic adaptation of the AUDIT questionnaire delivered by ACASI in the Kinyarwanda language and Rwandan context. This study also is the first to evaluate the psychometric properties of the AUDIT questionnaire using data from patients in the Rwandan healthcare setting. In Rwanda specifically, studies have been done using Kinyarwanda-translated versions of the AUDIT [17–20]. Yet, these studies have not provided the translated version or evidence of its validity or reliability. The exception to this includes two studies conducted by Kanyoni et al. and Harbertson et al. who reported a Cronbach’s Alpha of 0.808, and 0.79, respectively, but the translated AUDIT itself, adaptation methodology and further analyses were not reported [17,18]. In our study, we filled this gap in knowledge by exploring various measures of reliability and validity to provide evidence of a culturally adapted tool. The AUDIT questionnaire showed similar psychometric properties and performed as expected. Thus, due to Rwanda being one of the most densely population countries in the world with less urban/rural division, we believe the Kinyarwanda-translated AUDIT questionnaire can be used to evaluate alcohol use across the Rwandan healthcare system.

Our patient data findings indicate that the Rwanda-specific AUDIT questionnaire retains satisfactory concurrent validity, mirroring the original AUDIT questionnaire. The original AUDIT questionnaire was developed to include three structural dimensions to test an individual’s frequency of alcohol use, alcohol dependence, and risk behavior [7,8]. We employed CFA and assessed dimensionality, comparing one-dimensional, two-dimensional, and three-dimensional constructs of the AUDIT validation. Our findings affirm the construct’s one-dimensionality, as neither the two-dimensional nor three-dimensional models showed significant deviations from the one-dimensional model (Fig 2) Table 3. This finding is consistent with expectations based on the original AUDIT scale [7,8] and its cross-cultural variants in various regions, including findings across sub-Saharan Africa [13–16].

In addition to the excellent construct validity, the AUDIT questionnaire was shown to have strong concurrent validity. When scores of the adapted questionnaire were compared between drinkers and non-drinkers (Fig 3), the adapted version captured higher scores among drinkers and lower scores among non-drinkers. Additionally, the total AUDIT scores were correlated with total DSM scores, which showed a positive, linear correlation between the two (Fig 4). Thus, the adapted AUDIT maintains adequate construct validity.

The Kinyarwanda AUDIT questionnaire was found to have strong internal consistency. The reliability scores, including Cronbach’s Alpha, Omega, and Composite Reliability for each dimensional model, were all above 0.70 Table 4. The one-and-two-dimensional models performed better with reliability scores above 0.80. These results underscore that all three- dimensional models satisfy the internal consistency standards established by psychometric analyses [31,34]. Our results emphasize previous work in the region where the AUDIT questionnaire maintained strong internal consistency across sub-Saharan Africa [13–16].

Our study highlights that the adapted AUDIT screening questionnaire is culturally and linguistically validated to screen for AUD in the Rwandan context. Yet, this finding must be considered in the limitations of our study. First, our instrument showed excellent content and construct validity, and some evidence of concurrent validity based on its correlation with DSM-5 criteria administered via survey, however, it remains to be tested against the reference standard of a psychiatric clinical interview to assess for DSM-5 criteria. Likewise, our instrument showed excellent internal consistency, but it has not yet been tested for external reliability via a test-retest process. We only evaluated adults at an urban medical center. Further testing should validate this instrument among adolescents and patients from rural settings. Finally, additional research is needed to ensure the tool performs consistently between groups where alcohol consumption patterns differ.

## Conclusion

The Rwanda-focused AUDIT showed excellent performance for measures of internal structure and strong internal consistency. This evidence supports the AUDIT’s reliability as a screening instrument for patients at risk for alcohol-related harm in Rwanda. Although this study is the first step in addressing alcohol-related harm, future research should focus on evaluating the properties between groups where alcohol consumption patterns differ to capture those at risk in the population adequately. In Africa, and globally, the high and worsening impact of alcohol use calls for the critical need to adapt current screening tools. This study and adapted AUDIT tools will address this need and will be crucial to curbing this public health threat in Rwan

## Supporting information

S1 File Rwanda-focused AUDIT. (DOCX)

S2 File Rwanda-focused AUDIT standard drinks card. (PDF)
