# Supplementary material for: Adaptation and validation of a Rwanda-focused version of the Alcohol Use Disorder Identification Test (AUDIT)
Source: PLoS One. 2025 Feb 25;20(2):e0316993. doi: 10.1371/journal.pone.0316993 (PMC11856578; doi:10.1371/journal.pone.0316993)
Supplement: S1 File — (DOCX) [file pone.0316993.s001.docx]

**AUDIT**

|  | **FILE NAME** | ENGLISH AUDIO | KINYARWANDA AUDIO |
| --- | --- | --- | --- |
| 0 | AUDIT_00_INTRO | The following section will ask about your use of alcoholic beverages during the **past year**.    An alcoholic beverage includes beer like Primus and similar brand, homemade banana beer, store-bought banana beer like Akarusho and similar brands, and liquor such as Konyagi and similar brands.    When we say “standard drink” we mean the amount of alcohol in a small bottle of beer, like Primus. For stronger alcohol, a smaller amount equals one “standard drink” | Icyiciro gikurikira kirabaza uburyo wakoresheje ibinyobwa bisembuye mu mezi 12 ashize.  Ibinyobwa bisembuye harimo inzoga nka Primusi n’izindi bimeze kimwe, urwagwa  rutunganyirizwa mu rugo, inzoga y’urwagwa ikorwa n’inganda nk’akarusho n’izindi nka zo, na likeri twavuga nka Konyagi n’izindi bibarirwa hamwe.  Iyo tuvuze icupa fatizo tuba dusobanuye ingano y’alukoro iri mu icupa rito rya Primusi cg indi iri mu kirahure kiringaniye ku nzoga zikaze |
|  | STD_DRINKS_CARD | For beer like Primus there is **1** “standard drink” in a small (330 ml) bottle and there are **2** “standard drinks” in a large (720 ml) bottle  For Urwagwa, there is **1** “standard drink” in a medium glass  For Urwagwa Rukaze like Akarusho, there is **1** “standard drink” in a small glass and there are **4** “standard drinks” in 500 ml bottle  For liquor like Konyagi there is **1** “standard drink” in a very small glass, like a shot glass, and there are **10** “standard drinks” in 500 ml bottle | Ku nzoga nka Primusi, icupa 1 ritoya rya mililitiro 330 ringana n'icupa  fatizo” **1**. Icupa rinini rya mililitiro 720 ringana n'amacupa fatizo” **2**    Ku rwagwa, “Icupa fatizo” 1 ringana n’ikirahure **1** kiringaniye    Ku Urwagwa Rukaze nk’ Akarusho,  “Icupa fatizo” 1 ringana n’akarahure **1** gato. Icupa rya mililitiro 500 ringana n’amacupa fatizo **4**        Kuri likeri nka Konyagi, “icupa fatizo” **1** ringana n’akarahure 1 gato cyane bakunda kwita  godet. Icupa rya mililitiro 500 ringana n “amacupa fatizo” **10** |
| 1 | AUDIT_01 | How often do you have a drink containing alcohol?  For "Never ", press 0.  For "Monthly or less", press 1.  For "2 to 4 times a month ", press 2.  For "2 to 3 times a week", press 3.  For "4 or more times a week ", press 4. | Ni inshuro zingahe unywa ibinyobwa bisembuye? Niba ari nta na rimwe, kanda 0  Niba ari gake bitarenze rimwe mu kwezi kanda 1  Niba ari Inshuro 2 kugeza kuri 4mu kwezi kanda 2 Niba ari  Inshuro 2 kugeza kuri 3 mu cyumweru kanda 3 Niba ari  Inshuro 4 cyangwa zirenga mu cyumweru kanda 4 |
| 2 | AUDIT_02 | How many drinks containing alcohol do you have on a typical day when you are drinking?  For "1 or 2", press 0.  For "3 or 4", press 1.  For "5 or 6", press 2.  For "7, 8, or 9 ", press 3.  For "10 or more", press 4. | Ubusanzwe, iyo uri kunywa inzoga, unywa amacupa angahe?  Niba ari icupa 1 cyangwa 2 kanda 0  Niba ari amacupa 3 cyangwa 4 kanda 1 Niba ari amacupa 5 cyangwa 6 kanda 2 Niba ari amacupa 7, 8 cyangwa 9 kanda 3 Niba ari amacupa 10 cyangwa arenga kanda 4 |
| 3 | AUDIT_03 | How often do you have six or more drinks on one occasion?  For "Never", press 0.  For "Less than monthly ", press 1.  For "Monthly", press 2.  For "Weekly", press 3.  For "Daily or almost daily ", press 4. | Ni kangahe ushobora kunywa amacupa mato 6 cyangwa arenga mu gihe kimwe?  Niba ari nta na rimwe kanda 0 Niba ari gake bitari buri kwezi kanda1 Niba ari buri Kwezi kanda 2 Niba ari buri Cyumweru kanda 3 Niba ari buri munsi cyangwa hafi ya buri munsi kanda 4 |
| 4 | AUDIT_04 | How often during the last year have you found that you were not able to stop drinking once you had started?  For "Never", press 0.  For "Less than monthly ", press 1.  For "Monthly", press 2.  For "Weekly", press 3.  For "Daily or almost daily ", press 4. | Mu mezi 12 ashize, ni inshuro zingahe wabaga watangiye kunywa, ukiyumva udashoboye kubihagarika ubwawe? Niba ari nta na rimwe kanda 0 Niba ari gake. Bitari buri kwezi kanda 1 Niba ari buri Kwezi kanda 2 Niba ari buri Cyumweru kanda 3 Niba ari buri munsi cyangwa hafi ya buri munsi kanda 4 |
| 5 | AUDIT_05 | How often during the last year have you failed to do what was normally expected from you because of drinking?  For "Never", press 0.  For "Less than monthly ", press 1.  For "Monthly", press 2.  For "Weekly", press 3.  For "Daily or almost daily ", press 4. | Mu mezi 12 ashize, ni inshuro zingahe wananiwe kuzuza inshingano zawe bitewe no kunywa inzoga?  Niba ari nta na rimwe kanda 0 Niba ari gake bitari buri kwezi kanda1 Niba ari buri Kwezi kanda 2 Niba ari buri Cyumweru kanda 3 Niba ari buri munsi cyangwa hafi ya buri munsi kanda 4 |
| 6 | AUDIT_06 | How often during the last year have you needed a first drink in the morning to get yourself going after a heavy drinking session?  For "Never", press 0.  For "Less than monthly ", press 1.  For "Monthly", press 2.  For "Weekly", press 3.  For "Daily or almost daily ", press 4. | Ni inshuro zingahe mu mezi 12 ashize waba warakeneye kunywa inzoga mu gitondo kugira ngo ugarure imbaraga nyuma y'uko wari waherutse kunywa inzoga nyinshi? Niba ari nta na rimwe kanda 0 Niba ari gake bitari buri kwezi kanda1 Niba ari buri Kwezi kanda 2 Niba ari buri Cyumweru kanda 3 Niba ari buri munsi cyangwa hafi ya buri munsi kanda 4 |
| 7 | AUDIT_07 | How often during the last year have you had a feeling of guilt or remorse after drinking?  For "Never", press 0.  For "Less than monthly ", press 1.  For "Monthly", press 2.  For "Weekly", press 3.  For "Daily or almost daily ", press 4. | Ni inshuro zingahe mu mezi 12 ashize, wumvise ugize umutima ukurega cyangwa wicuza nyuma yo kunywa inzoga?  Niba ari nta na rimwe kanda 0 Niba ari gake bitari buri kwezi kanda1 Niba ari buri Kwezi kanda 2 Niba ari buri Cyumweru kanda 3 Niba ari buri munsi cyangwa hafi ya buri munsi kanda 4 |
| 8 | AUDIT_08 | How often during the last year have you been unable to remember what happened the night before because you had been drinking?  For "Never", press 0.  For "Less than monthly ", press 1.  For "Monthly", press 2.  For "Weekly", press 3.  For "Daily or almost daily ", press 4. | Ni inshuro zingahe mu mezi 12 ashize, wananiwe kwibuka ibyabaye mu ijoro ryabanje, bitewe n'uko wari wanyoye?  Niba ari nta na rimwe kanda 0 Niba ari gake bitari buri kwezi kanda1 Niba ari buri Kwezi kanda 2 Niba ari buri Cyumweru kanda 3 Niba ari buri munsi cyangwa hafi ya buri munsi kanda 4 |
| 9 | AUDIT_09 | Have you or someone else been injured as a result of your drinking?  For "No", press 0.  For "Yes, but not in the last year ", press 2.  For "Yes, during the last year ", press 4. | Hari ubwo wowe cyangwa undi muntu yaba yarakomeretse bitewe n’uko wari wanyoye?  Niba ari Oya kanda 0  Niba ari  Yego, ariko atari mu mezi 12 ashize kanda 2 Niba ari Yego, mu mezi 12 ashize  kanda 4 |
| 10 | AUDIT_10 | Has a relative or friend or a doctor or another health worker been concerned about your drinking or suggested you cut down?  For "No", press 0.  For "Yes, but not in the last year ", press 2.  For "Yes, during the last year ", press 4. | Ese hari uwo mu muryango wawe, inshuti, umuganga cyangwa undi ukora mu by’ubuvuzi, wigeze ahangayikishwa n'uburyo unywa inzoga cyangwa akagusaba kubigabanya?  Niba ari Oya kanda 0  Niba ari  Yego, ariko atari mu mezi 12 ashize kanda 2 Niba ari Yego, mu mezi 12 ashize  kanda 4 |
