# Supplementary material for: Adaptation and validation of a Rwanda-focused version of the Alcohol Use Disorder Identification Test (AUDIT)
Source: PLoS One. 2025 Feb 25;20(2):e0316993. doi: 10.1371/journal.pone.0316993 (PMC11856578; doi:10.1371/journal.pone.0316993)

English

Inzoga (Urugero, Primus)
3-6% Alukolu

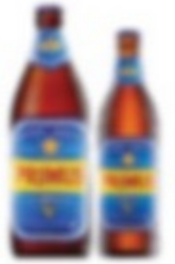

There is **1** “standard drink” in a small (330 ml) bottle

There are **2** “standard drinks” in a large (720 ml) bottle

Urwagwa
6-10% Alukolu

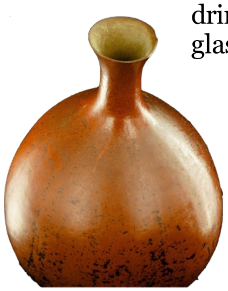

There is **1** “standard drink” in a medium glass

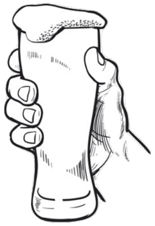

Urwagwa Rukaze (Urugero, Akarusho)
10-15% Alukolo

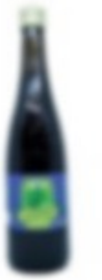

There is **1** “standard drink” in a small glass

There are **4** “standard drinks” in 500 ml bottle

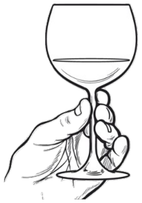

Likeri (Urugero, Konyagi)
Hejuru ya 15% Alukolo

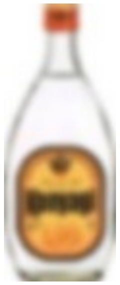

There is **1** “standard drink” in a very small glass (shot glass)

There are **10** “standard drinks” in 500 ml bottle

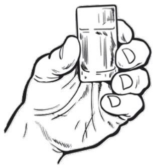

Kinyarwanda

Inzoga (Urugero, Primus)
3-6% Alukolu

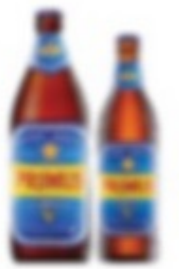

Mu icupa 1 ritoya rya mililitiro 330 harimo "icupa fatizo" **1.**

Mu icupa rinini rya mililitiro 720 har imo "amacupa fatizo" **2**

Urwagwa
6-10% Alukolu

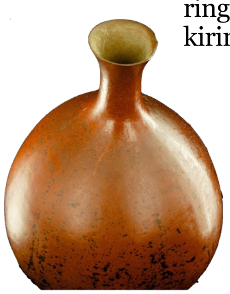

"Icupa fatizo" 1 ringana n'ikirahure [illegible] kiringaniye

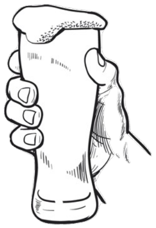

Urwagwa Rukaze (Urugero, Akarusho)
10-15% Alukolo

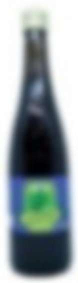

"Icupa fatizo" 1 ringana n'akarahure **1** gato

Icupa rya mililitiro 500 ringana n'amacupa fatizo **4**

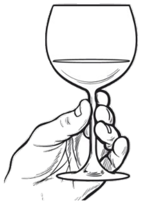

Likeri (Urugero, Konyagi)
>15% Alukolo

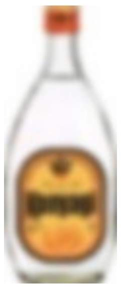

"icupa fatizo" **1** ringana n'akarahure gato cyane (godet)

Icupa rya mililitiro 500 ringana n "amacupa fatizo **10**

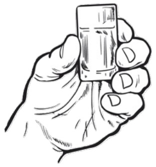

Supplement: S2 File — (PDF) [file pone.0316993.s002.pdf]
